# Efficacy of Polynucleotide‐Based Therapy in Atopic Dermatitis Management

**DOI:** 10.1111/jocd.70648

**Published:** 2026-01-02

**Authors:** Kyungtae Bae, Youkyoung Cho, Kookjee Soo, Jeongwoo Lee, Youngjin Park, Yoonjeung Hyun, Jin‐Hyun Kim, Kyuho Yi

**Affiliations:** ^1^ It's Me Clinic Sejong Republic of Korea; ^2^ CHA University School of Medicine Pocheon Republic of Korea; ^3^ ELEV Clinic Seoul Republic of Korea; ^4^ Obliv Clinic Seoul Republic of Korea; ^5^ Yonseifams Clinic Seoul Republic of Korea; ^6^ You and I Clinic Seoul Republic of Korea; ^7^ Division in Anatomy and Developmental Biology, Department of Oral Biology Human Identificaton Research Institute, BK21 Four Project, Yonsei University College of Dentistry Seoul Korea

**Keywords:** anti‐inflammatory, atopic dermatitis, injectable therapy, polynucleotide, skin barrier, skin hydration, skin regeneration

## Abstract

**Background:**

Atopic dermatitis (AD) is a chronic inflammatory skin disease characterized by persistent itching, dryness, redness, and recurrent eczematous lesions, significantly impacting patients' quality of life and psychological well‐being. Despite various effective conventional treatments, innovative therapeutic approaches that simultaneously provide anti‐inflammatory effects and enhanced skin hydration remain essential.

**Objective:**

This case‐series study aimed to evaluate the clinical efficacy, patient satisfaction, and safety profile of polynucleotide (PN)‐based injectable therapies in patients with AD.

**Methods:**

Patients with clinically diagnosed AD underwent intradermal injections of PN. Treatment outcomes were assessed by comparing standardized clinical photographs taken before and after the PN treatment, focusing on improvements in erythema, skin thickness, and overall skin hydration. Additionally, patient‐reported outcomes, including reductions in itching, discomfort, and treatment satisfaction, were evaluated.

**Results:**

Posttreatment evaluations demonstrated notable clinical improvements, including reduced erythema, decreased skin thickness, and enhanced skin hydration and elasticity. Patients consistently reported significant alleviation of itching and overall discomfort. Furthermore, high patient satisfaction and good adherence to treatment were observed, with no significant adverse events or side effects reported.

**Conclusion:**

This exploratory case series provides preliminary observations suggesting that PN‐based injectable therapies may offer benefits for patients with AD, particularly in improving skin hydration, barrier function, and symptomatic relief. While positive outcomes were documented in all four cases, the absence of validated scoring systems, objective biophysical measures, long‐term follow‐up, and a control group limits the strength and generalizability of these findings. PN injections in this context remain off‐label, and further prospective, controlled studies with larger, more diverse populations are essential to establish efficacy, safety, optimal dosing regimens, and durability of response.

## 1 Introduction

Atopic dermatitis (AD) is a chronic inflammatory skin disease characterized by persistent itching, dryness, redness, and recurrent eczematous lesions [1]. AD significantly compromises patient quality of life, causing physical discomfort, sleep disturbances, emotional distress, social isolation, and reduced overall life satisfaction. The burden of AD frequently extends beyond physical symptoms, markedly affecting patients' mental well‐being and daily functioning, particularly in moderate to severe cases [2, 3, 4].

The pathogenesis of AD involves a complex interplay of genetic predisposition, immune dysregulation, and impaired epidermal barrier function, resulting in increased transepidermal water loss, altered skin microbiota, and chronic inflammatory responses predominantly driven by cytokines such as IL‐4, IL‐13, and IL‐31 [5]. Current management strategies for AD include topical corticosteroids, calcineurin inhibitors, systemic immunomodulators, and supportive skincare regimens designed to restore epidermal barrier function [5]. Although generally effective, continuous exploration of innovative therapies is necessary to fulfill diverse patient needs. Specifically, treatments offering concurrent anti‐inflammatory effects and enhanced hydration may further broaden the therapeutic options available for AD patients [6, 7, 8].

Recently, polynucleotide (PN)‐based injectable therapies, biologically active molecules derived from salmon DNA, have attracted attention due to their multifaceted regenerative and anti‐inflammatory capabilities. PN exerts its effects through several mechanisms relevant to AD pathophysiology. Notably, PN interacts with adenosine A2A receptors, modulating inflammation by reducing cytokine production, including TNF‐α and IL‐6 [6, 7, 8]. While these cytokines are not central mediators in the Th2‐type inflammatory response characteristic of AD, their suppression may still contribute positively by alleviating chronic inflammation, potentially mitigating secondary exacerbations, and reducing skin barrier impairment.

AD is primarily driven by a Th2‐skewed immune response, with cytokines such as IL‐4 and IL‐13 playing central roles in disease pathogenesis. PN‐based therapies are not proposed as direct modulators of these canonical Th2 pathways. Rather, they may act indirectly by supporting skin barrier integrity, improving dermal microenvironmental conditions, and reducing non‐Th2 inflammatory tone through mechanisms such as attenuation of TNF‐α and IL‐6. These potential effects should therefore be considered complementary to, rather than substitutive for, established therapeutic strategies that specifically target the Th2 axis.

A study has further demonstrated the therapeutic potential of adenosine receptor activation in allergic conditions, including asthma, through the modulation of immune responses and enhancement of regulatory T‐cell populations [9]. Given the immunological overlap between asthma and AD, PN's ability to activate adenosine A2A receptors might similarly help control chronic inflammation in AD skin. Specifically, by reducing pro‐inflammatory cytokine levels and promoting immune tolerance via regulatory T cells, PN‐based therapies may represent a novel adjunctive approach to managing inflammation and immune dysregulation in AD [6, 7, 8].

In addition to these anti‐inflammatory actions, PN demonstrates notable regenerative effects, including the promotion of angiogenesis and extracellular matrix remodeling, primarily through increased expression of vascular endothelial growth factor (VEGF) and stabilization of newly formed vessels [10]. Furthermore, PN promotes fibroblast proliferation and collagen synthesis, enhancing skin repair and supporting epidermal integrity [8]. PN molecules also participate in nucleotide salvage pathways, supplying nucleotides essential for DNA synthesis and cellular repair, which can significantly enhance cellular regeneration in chronically inflamed and repeatedly injured atopic skin [8].

A further beneficial characteristic of PN is its intrinsic water‐binding capacity, significantly improving skin hydration and elasticity. Although hyaluronic acid (HA) has traditionally been utilized to improve skin moisture retention in inflammatory skin disorders, recent comparative studies suggest that PN may offer superior hydration effects. Clinical trials have demonstrated greater improvements in skin hydration, elasticity, erythema, and reduced roughness following PN injections compared to HA, underscoring PN's potential efficacy in addressing AD‐associated dryness [11].

Given these promising biological rationales and preliminary supportive evidence, further clinical investigations evaluating PN therapy specifically in AD are warranted. Currently, structured clinical data directly assessing the clinical efficacy, safety, and patient‐reported outcomes associated with PN use in AD remain limited.

This study aims to explore the practical clinical outcomes of PN therapy for AD and contribute foundational evidence supporting further clinical application and investigation. By highlighting detailed patient outcomes and providing practical clinical insights, this research seeks to illustrate the potential role of PN‐based therapies in AD management, laying the groundwork for further clinical exploration and validation.

## 2 Methods

### 2.1 Ethics and Consent

All patients provided written informed consent for the off‐label use of PN injections and for the publication of de‐identified clinical data and images.

### 2.2 Patient Selection

Patients presenting with AD partially responsive to conventional therapies were selected. Inclusion criteria comprised AD location, size (approximately palm‐size area per treated region), and patient willingness to undergo PN‐based injectable therapy.

During the treatment period, no additional active therapies for AD were permitted, including topical corticosteroids, calcineurin inhibitors, or systemic immunomodulators. Patients continued only with routine use of bland emollients for basic skin maintenance, with no modifications introduced during the study window. This restriction was applied to minimize confounding influences and to allow clearer attribution of observed clinical changes to the PN injections.

### 2.3 PN Treatment Protocol

PN injections (Vitaran, BR Pharm Co. Ltd., South Korea) were administered intradermally at a concentration of 2 cc per palm‐sized scar area. The treatment regimen consisted of four sessions spaced at 3 week intervals.

Intradermal injections of PN were performed using a 33‐gauge, 4 mm needle. The average injection volume per point was approximately 0.02–0.05 mL, administered at 1 cm intervals across the affected area. The injection depth was limited to the dermis, with care taken to avoid overfilling and intravascular placement. This standardized technique was applied consistently across all treatment sessions to ensure reproducibility and clinical consistency.

### 2.4 Evaluation Method

Treatment outcomes were assessed using standardized clinical photography taken before and after treatment. Photographic documentation was compared to evaluate improvements in skin appearance and hydration.

### 2.5 Safety and Patient Satisfaction

Safety assessments included monitoring for adverse events and patient‐reported discomfort. Patient satisfaction was assessed verbally at follow‐up visits.

### 2.6 Evaluation

Treatment outcomes were assessed primarily through observational clinical photography and patient‐reported impressions. Standardized procedures were used to the extent possible, including consistent camera equipment, fixed distance, identical angles, and controlled lighting conditions at each visit. Images were compared before and after the treatment series to document visible changes in erythema, skin thickness, and hydration. No validated eczema severity indices (such as EASI, SCORAD, or POEM) or objective biophysical measures (such as transepidermal water loss or corneometry) were collected, reflecting the exploratory and descriptive nature of this case series.

### 2.7 Treatment Protocol Justification

The dosing regimen of approximately 2 cc per palm‐sized area at intervals of 2–3 weeks was selected on pragmatic clinical grounds. This volume allowed for consistent intradermal micro‐depot placement, promoting even dermal distribution while minimizing the risk of excessive oedema or procedural discomfort. The spacing of injection points at roughly 1 cm intervals followed common microinjection practices in dermatologic procedures, facilitating reproducibility across sessions. However, this approach remains empirical within the feasibility context of this case series, and no formal dose‐ranging data are currently available. As noted in the Discussion, optimization of dose, frequency, and interval requires systematic evaluation in controlled trials.

## 3 Case Reports

### 3.1 Case 1

A 28 year‐old female patient presented with AD located at the popliteal fossa and antecubital fossa. Prior to treatment initiation on October 19, 2024, the AD exhibited significant erythema, thickness, and rigidity, causing discomfort, including itching, as well as aesthetic dissatisfaction.

**FIGURE 1:**
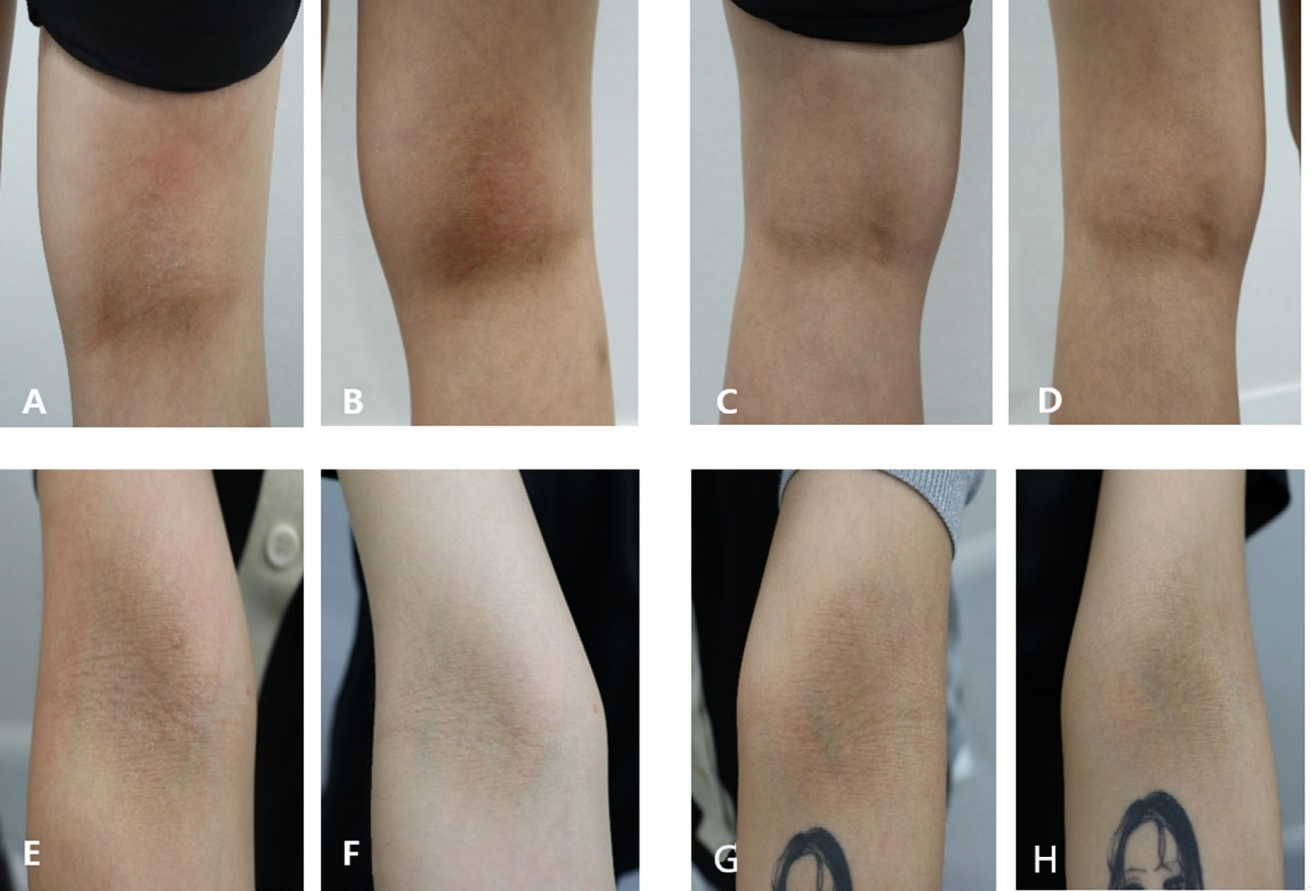
Pre and posttreatment images demonstrate atopic dermatitis at the popliteal fossa exhibiting erythema, thickness, and rigidity before treatment. After completing a series of sessions, substantial improvements in appearance and hydration were observed, highlighting the therapy's effectiveness in managing atopic dermatitis. Images A and B: Pre and posttreatment of the right side of the popliteal fossa. Images C and D: Pre and posttreatment of the left side of the popliteal fossa. Images E and F: Pre and posttreatment of the right side of the antecubital fossa. Images G and H: Pre and posttreatment of the left side of the antecubital fossa.

Following treatment with intradermal Vitaran (BR Pharm Co. Ltd., South Korea) injections (2 cc per area per session), noticeable improvements were observed by December 28, 2024. Posttreatment photographic evaluation revealed marked reductions in erythema, notable decreases in skin thickness, and improved hydration (Figure 1). Visually, the treated areas appeared smoother and less inflamed, contributing to enhanced comfort and reduced itching. Patient‐reported satisfaction was high, and the procedure was well‐tolerated, with no adverse events or side effects noted.

### 3.2 Case 2

A 23‐year‐old female patient with eczema in an atopy‐prone individual located at the heel area presented for treatment. Initial evaluation on November 7, 2024, showed a thickened, rigid, and erythematous dermatitis contributing to functional discomfort during ambulation.

**FIGURE 2:**
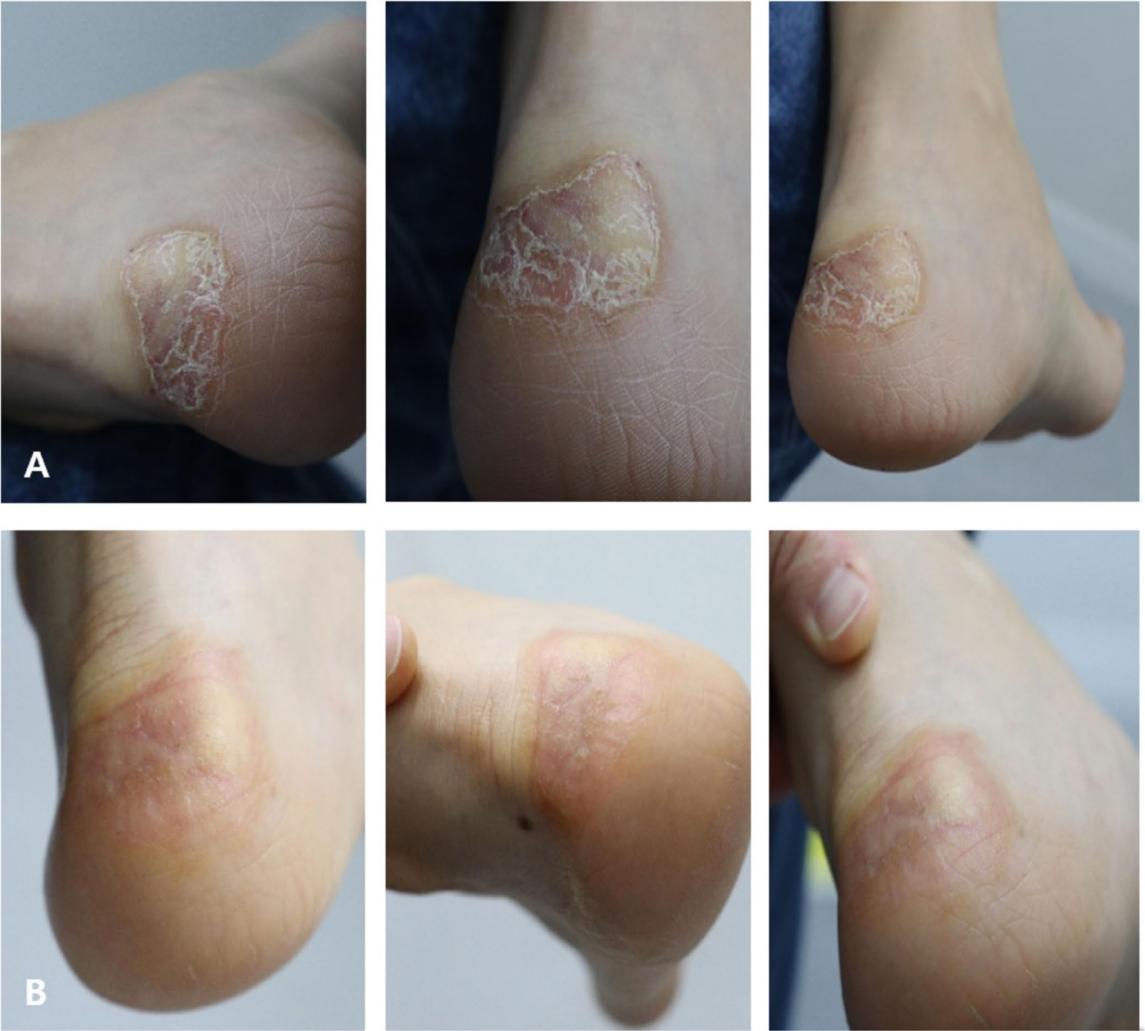
Pre and posttreatment images demonstrate the atopic dermatitis at the heel exhibiting thickness, erythema, and reduced elasticity before treatment. After completing a series of sessions, substantial improvements in appearance, hydration, and elasticity are observed, highlighting the effectiveness of the therapy for managing eczema in an atopy‐prone individual at challenging sites. Images A and B: Pre and posttreatment of heel.

After receiving intradermal Vitaran (BR Pharm Co. Ltd., South Korea) injections (2 cc per area per session), substantial improvements were evident by December 23, 2024. Photographic assessment posttreatment demonstrated visibly reduced thickness and decreased erythema of the treated area, alongside significantly improved skin hydration and flexibility (Figure 2). The patient reported increased comfort during ambulation and expressed high satisfaction with the treatment outcome. No adverse reactions or complications were encountered.

### 3.3 Case 3

A 28 year‐old female (born in 1996) presented with AD affecting the dorsal aspect of both hands, characterized by erythema, dryness, and intermittent itching. Despite previous use of topical corticosteroids and moisturizers, symptoms persisted.

She underwent intradermal injection of 2 cc of Vitaran (BR Pharm Co. Ltd., South Korea) at each session, performed three times at 2 week intervals. Photographs were taken immediately before the first session and 2 weeks following the final treatment.

**FIGURE 3:**
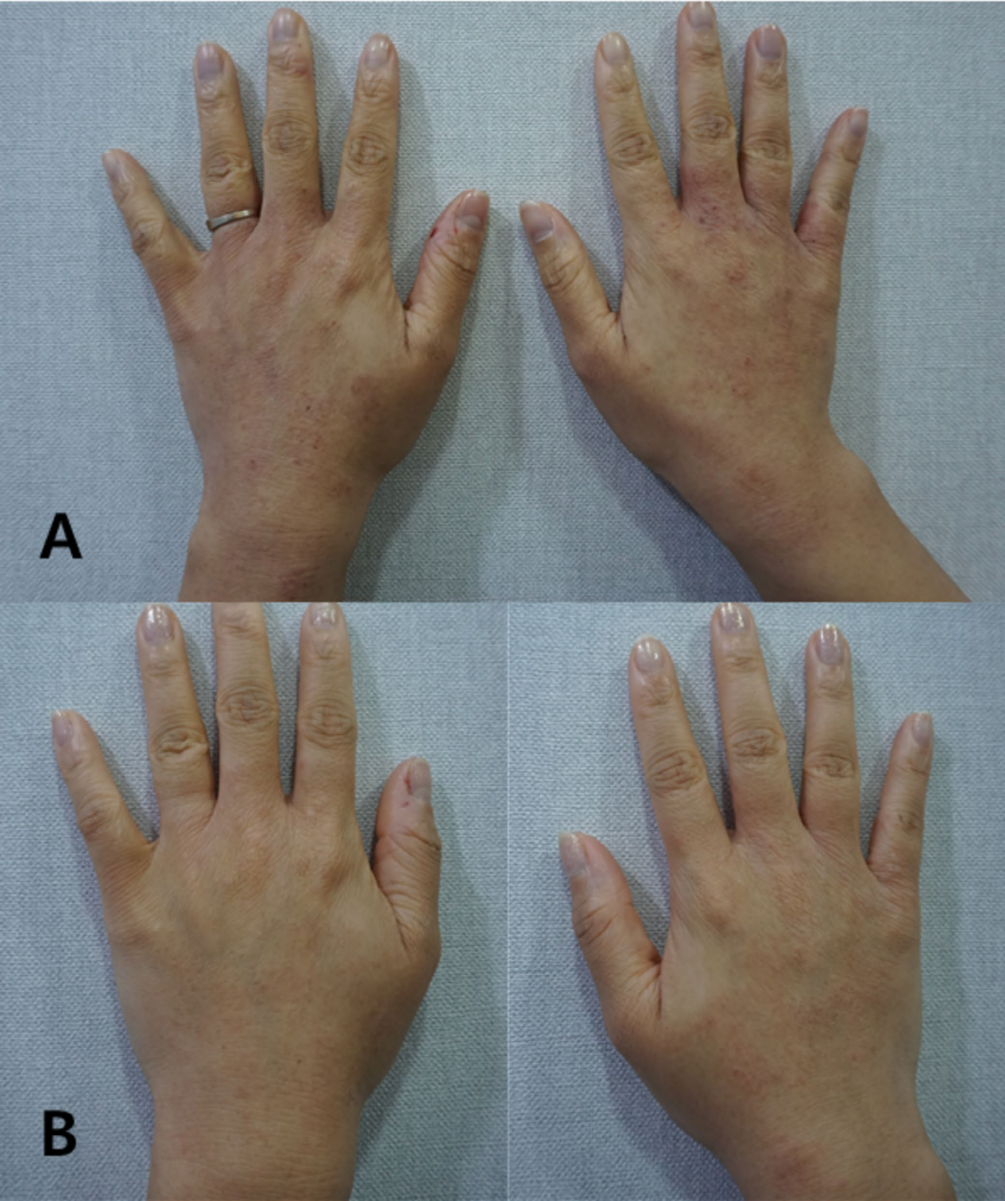
Atopic dermatitis on the dorsal hand of a 28 year‐old female (Case 3). Panel A: Pretreatment photo showing erythema, scaling, and thickened texture. Panel B: Two weeks after the final injection, demonstrating clear clinical improvement in inflammation and surface texture.

Posttreatment evaluation demonstrated notable improvement, including reduced erythema, smoother skin texture, and restored hydration. The patient reported substantial relief from itching and expressed high satisfaction. No adverse events were observed (Figure 3).

### 3.4 Case 4

A 35 year‐old female patient (born in 1989) presented with chronic AD on the dorsal hand, marked by persistent erythema, dryness, and pruritus refractory to conventional topical treatments.

**FIGURE 4:**
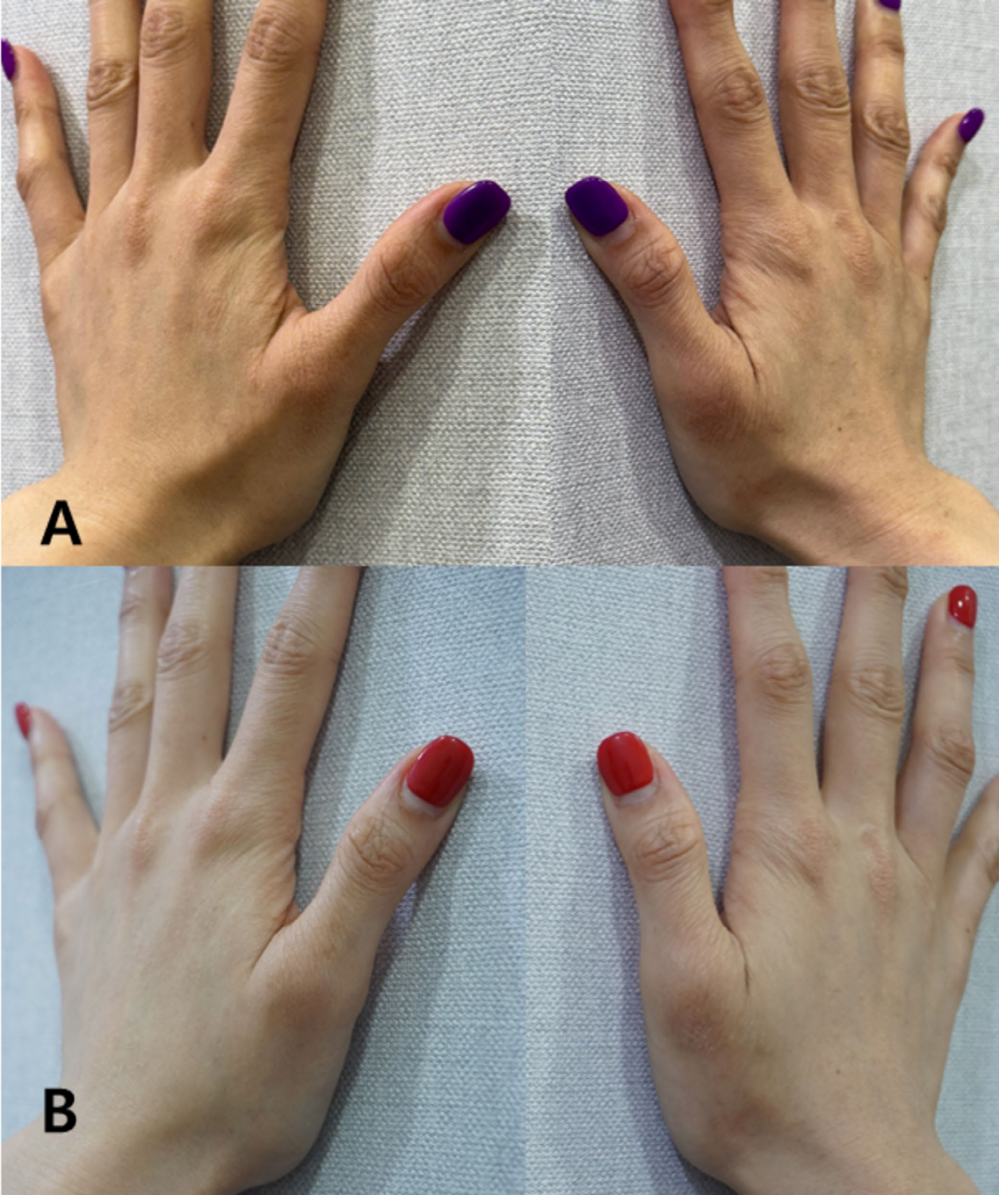
Atopic dermatitis on the dorsal hand of a 35 year‐old female patient. Pre and posttreatment images demonstrate erythema, dryness, and rough texture before treatment. After completing three sessions of intradermal injections (2 cc per session, 2 week intervals), substantial improvement was observed, including reduced erythema and smoother skin texture. Images A and B: Pre and posttreatment of the right dorsal hand.

She received 2 cc of intradermal Vitaran (BR Pharm Co. Ltd., South Korea) administered at each session, with three total sessions spaced 2 weeks apart. Standardized clinical photographs were obtained immediately before the first session and again 2 weeks following the final treatment (Figure 4).

The follow‐up evaluation revealed visible clinical improvement, including diminished erythema, smoother skin texture, and reduced dryness. The patient reported reduced itching and increased comfort in daily activities. No adverse reactions or complications were noted.

## 4 Discussion

This case‐series study aimed to evaluate the clinical effectiveness of PN‐based injectable therapies in managing AD. Based on clinical observations from the cases included, intradermal injections of PN demonstrated noticeable improvements in skin erythema, thickness, hydration, and overall skin quality. Patients reported visible relief from AD‐associated itching and discomfort, accompanied by high levels of treatment satisfaction and adherence. Importantly, this study provides early evidence regarding the potential therapeutic role of PN in AD, a condition previously not extensively evaluated within the context of PN therapies.

The clinical benefits observed in these cases can be explained by several established biological mechanisms of PN relevant to AD pathophysiology. PN interacts with adenosine A2A receptors, exerting anti‐inflammatory effects by modulating pro‐inflammatory cytokines [6, 7, 8]. Although these cytokines are not central to the Th2‐driven inflammatory response characteristic of AD, their reduction may beneficially alleviate chronic inflammatory conditions, potentially preventing secondary exacerbations and assisting in barrier restoration [5, 10, 12]. Additionally, the activation of A2A receptors may promote immune tolerance and regulatory T cell responses, potentially offering further immunomodulatory advantages in AD management [9].

Furthermore, PN enhances skin regeneration and barrier restoration by stimulating fibroblast proliferation, collagen synthesis, and angiogenesis through increased VEGF expression [6, 8, 10]. Such effects are critical for supporting epidermal integrity and facilitating skin repair processes frequently compromised in AD. Additionally, PN molecules participate in the nucleotide salvage pathway, providing nucleotides necessary for DNA repair and cellular regeneration, further promoting resilience in chronically inflamed and frequently injured atopic skin [8].

An essential and distinctive advantage of PN therapy observed in this study was its intrinsic water‐binding property, contributing significantly to improved skin hydration and elasticity. These hydration benefits may represent a considerable therapeutic advantage in managing AD, given that impaired skin barrier function and persistent dryness are hallmark features of the disease. Indeed, recent comparative clinical research indicates that PN may outperform traditional hydration agents, such as HA, in enhancing skin moisture retention, elasticity, and overall skin texture [11].

Previous studies have supported the beneficial biological effects of PN across various dermatological conditions. Animal studies demonstrated the anti‐inflammatory, regenerative, and angiogenic capabilities of PN through enhanced VEGF expression, improved wound healing, and reduced inflammatory cell infiltration [10, 12]. Clinical studies in other inflammatory skin conditions and aesthetic dermatology contexts have similarly supported PN's efficacy in improving hydration and visual erythema reduction [13, 14, 15]. Collectively, these prior findings provide additional support for the positive clinical outcomes observed in our AD cases.

Despite the encouraging results presented, several limitations must be acknowledged. Primarily, the small sample size of this case series significantly limits the generalizability of our findings. AD is inherently heterogeneous, with wide interindividual variability in disease severity, underlying pathophysiological mechanisms, and response to treatments. Therefore, predicting universally consistent clinical outcomes remains challenging. Additionally, this study focused on short‐term clinical responses, providing limited insight into long‐term efficacy and safety profiles associated with repeated PN administration. Furthermore, the evaluation was restricted to specific manifestations of AD, limiting conclusions about PN's effectiveness across diverse patient populations with varying degrees of severity and clinical presentations.

Nevertheless, this study holds important implications, providing foundational insights into the potential therapeutic role of PN‐based injectable therapies in AD management. Detailed clinical documentation, including high patient satisfaction, relief from itching and discomfort, and visible improvements in hydration and skin appearance, highlights the feasibility and acceptability of PN treatments in routine clinical practice. By introducing preliminary clinical data on PN efficacy specifically in AD, this research may inform larger, controlled studies aimed at validating treatment efficacy and establishing standardized clinical protocols.

The biological rationale for PN in AD is grounded in its known regenerative and hydration‐promoting properties, including stimulation of fibroblast proliferation, extracellular matrix remodeling, angiogenesis, and intrinsic water‐binding capacity. These mechanisms are consistent with the need for barrier repair and symptomatic relief in AD, particularly in relation to dryness, pruritus, and chronic barrier disruption. However, it must be acknowledged that much of the evidence supporting these actions derives from studies in wound healing, scar management, and aesthetic dermatology, rather than AD‐specific investigations.

This study has several important limitations. The small sample size, restricted to four young female patients, limits generalizability and does not reflect the heterogeneity of AD across different ages, sexes, and disease severities. The absence of a control or comparator group, reliance on nonstandardized photographic assessments, and use of subjective patient feedback without validated scoring instruments (e.g., EASI, SCORAD, POEM) or objective biophysical measures (e.g., TEWL, corneometry) reduce the robustness of the findings. The empiric dosing regimen (2 cc per palm‐sized area every 2–3 weeks) was not formally justified, and concomitant use of emollients or topical corticosteroids may represent confounding factors. In addition, most of the mechanistic rationale is extrapolated from wound‐healing and aesthetic contexts rather than AD‐specific studies, and follow‐up was short‐term, offering no insight into the durability of effect or relapse rates. Finally, the consistently positive outcomes raise the possibility of selection or reporting bias, particularly given the off‐label use of PN injections in this setting. These cases should therefore be viewed as exploratory observations intended to generate hypotheses and inform the design of larger, controlled trials with standardized and validated outcome measures.

Future studies should adopt a prospective, randomized, assessor‐blinded design comparing PN injections with standard care or HA, using validated outcome measures such as EASI (primary), POEM, TEWL, and corneometry (secondary), with longer follow‐up to assess durability and relapse rates at 12–24 weeks.

## 5 Conclusion

This case series underscores the promising clinical benefits of PN‐based injectable therapies for AD improvement. The positive clinical outcomes, high patient satisfaction, and preliminary evidence highlighting improvements in skin hydration, inflammation control, and symptom relief substantiate the therapeutic potential of PN therapies in managing AD, suggesting important avenues for further investigation and clinical innovation.

## Author Contributions

Kyungtae Bae and Youkyoung Cho: writing. Kookjee Soo3, Jeongwoo Lee, Youngjin Park, and Yoonjeung Hyun: writing review and editing. Jin‐Hyun Kim, Kyuho Yi: visualization. Kyu‐Ho Yi: supervision.

## Conflicts of Interest

The authors declare no conflicts of interest.

## Data Availability

The data that support the findings of this study are available from the corresponding author upon reasonable request.
